# SoxB, cell cycle and neurogenesis

**DOI:** 10.3389/fphys.2013.00298

**Published:** 2013-10-17

**Authors:** Theodoros Karnavas, Nikolaos Mandalos, Stavros Malas, Eumorphia Remboutsika

**Affiliations:** ^1^Stem Cell Biology Laboratory, Biomedical Sciences Research Centre “Alexander Fleming”Vari-Attica, Greece; ^2^The Cyprus Institute of Neurology and GeneticsNicosia, Cyprus

**Keywords:** Sox, stem cells, neural stem cells, neural progenitors, neurons, specification, commitment

*Sox* genes encode for a group of transcription factors that share the presence of an HMG-type (high mobility group) box. This is a 79 amino acid motif that encodes their DNA binding domain. They were identified soon after the discovery of the mammalian testis-determining gene *Sry*, which also possesses an HMG box, and obtained their name from *Sry* (“*Sox*” Sry-related box genes). The members of the Sox family are highly conserved among vertebrates and so far 26 different genes have been identified (Schepers et al., 2002; Wilson and Koopman, 2002). Sox proteins activate or repress target gene transcription, via interactions with a partner factor in a cell-type specific manner (Kondoh and Kamachi, 2010). Based on sequence homology, within and outside the HGM box, they have been classified into seven groups (SoxA to SoxG groups) (Wilson and Koopman, 2002).

*SoxB* genes, divided in *SoxB1 (Sox1-3*) and *SoxB2 (Sox14* and *21*) subgroups, code for a group of highly related transcription factors expressed mainly in the developing and adult Central Nervous System (CNS) in mammals (Wood and Episkopou, 1999; Schepers et al., 2002; Avilion et al., 2003; Sarkar and Hochedlinger, 2013). During embryonic development, the nervous system arises from primitive neuroepithelial cells that give rise to neural stem cells (NSCs), cells that have the potential to self-renew and differentiate into neurons, astrocytes and oligodendrocytes. Neuroepithelial progenitors (NEP) represent the cells with a more restricted potential, while precursors describe cells that exist in an earlier developmental state than others (McKay, 1997). In the developing CNS, *Sox1-3* and *Sox21* are expressed both in embryonic and adult NEPs and in subsets of differentiated neurons of the adult brain and spinal cord, whereas *Sox14* is expressed only in selected differentiated neurons of the embryonic and adult CNS. Proteins encoded by *Sox14* and *Sox21* genes are very similar to each other but distinct from those encoded by *Sox1-3*, in areas outside the HMG domain and Group B homology that lies immediately C-proximal of the HMG domain (Uchikawa et al., 1999). Sox1-3 proteins are thought to function as transcriptional activators, while Sox14/21 are believed to function as repressors. However, Sox1 and Sox2 have been shown to be involved in repression of transcription as well (Cavallaro et al., 2008; Elkouris et al., 2011). Despite the wide distribution in the expression of SoxB proteins in the developing CNS, the effort to unravel their distinct function has been hampered by redundancy issues, eventually leading to functional compensation, but insights into the function of each factor have been gained from studies in areas where unique expression of a single factor is detected.

SoxB function has been studied extensively during neurogenesis, the process of neuron formation, which involves continuous cycles of proliferation and differentiation of NEPs. During CNS development, dividing NEPs, residing in the ventricular zone (VZ), either self-renew or exit the cell cycle and differentiate. NEPs make cell fate decisions controlled by extrinsic and/or intrinsic determinants (Ramasamy et al., 2013). Neurons are generated in two sequential steps. In the specification step, which takes place within the VZ, some NEPs up-regulate the Notch receptors and remain as uncommitted progenitors while others down-regulate Notch receptors, up-regulate Notch ligands and then the proneural bHLH genes, Neurogenins 1-3 (Ngn1-3)/Mash1 and progress to terminal differentiation (Guillemot, 2007; Martynoga et al., 2012; Alberi et al., 2013). Accordingly, loss of Notch signaling invariably leads to over-production of neurons and depletion of NEPs (Pierfelice et al., 2011). In the commitment step, which is a transitionary phase and takes place on the lateral margins of the VZ known as intermediate zone (IZ), specified neuronal progenitors switch on another class of bHLH factors like NeuroD, Prox1, and Nscl1, which consolidate the specification step and allow the cells to progress to terminal differentiation (Christie et al., 2013). Ectopic expression of any of the latter can by-pass the specification phase and drive NEPs out of the cell cycle.

The transcription factors Sox1-3 have been proposed to block neuronal commitment and thus prevent differentiation (Bylund et al., 2003; Holmberg et al., 2008; Oosterveen et al., 2013). In contrast Sox21 opposes Sox1-3 function in NEPs, where they are co-expressed, and promotes neurogenesis *in vivo* (Sandberg et al., 2005). Given that SoxB1 and Sox21 factors are co-expressed in the NEPs and have been shown to bear antagonizing activities, there was an open question how a cell decides to remain uncommitted or exit the cell cycle and how the antagonizing function of these factors is modulated. One model would propose that the relative activities of Sox1-3 vs. Sox21 are thought to determine whether a cell will remain undifferentiated or commit to the neuronal lineage. Accordingly, loss of Sox21 could lead to a predominance of SoxB1 activity leading to a block on neurogenesis in the embryo. However, this is not the case during development, since loss of Sox21 leads to a block in the progression of neuronal specification in the hippocampus only in adult mice (Matsuda et al., 2012). For Sox1, the data had also been conflicting. *In vitro* studies suggested that Sox1, like Sox21, could promote neurogenesis while Sox2 or Sox3 can block it. Initial reports suggested that over-expression of Sox1, but not Sox2 or Sox3, could promote neurogenesis when over-expressed in primary cultures of neural stem/progenitor cells cultured as neurospheres and the carcinoma cell line P19 (Pevny et al., 1998; Kan et al., 2004, 2007). However, this model was not supported by *in vivo* loss-of-function analysis in mice. In contrast, the role of Sox1 has been best studied during neuronal differentiation in the developing ventral forebrain and spinal cord, where a unique role has been assigned during neuronal subtype specification (Ekonomou et al., 2005; Panayi et al., 2010). We believe that light into the role of SoxB genes during neuronal specification is likely to be shed by the results from double mutant experiments. For instance, despite the fact that *Sox1* and *Sox21* single mutant mice are born, double *Sox1/Sox21* mutants are embryonic lethal. The cause of lethality is yet to be determined (SM, unpublished data). Neuronal fates are determined around the final cell cycle of NEPs during which neural differentiation factors regulate the cell cycle either directly or indirectly. Only recently, SoxB genes have been implicated in the regulation of cell cycle. Studies in the mouse developing cortex showed that Sox1 acts to maintain the undifferentiated state of NEPs via a mechanism involving suppression of Prox1–mediated cell cycle exit and neurogenesis (Elkouris et al., 2011). These results are consistent with previous data from gain-of-function and loss-of-function analysis in the chick spinal cord, according to which SoxB1 factors block neurogenesis downstream of proneural factors (Bylund et al., 2003; Holmberg et al., 2008).

Sox2 has found itself in the position of the most celebrated of the Sox proteins due to its role in epiblast formation (Avilion et al., 2003; Mandalos et al., 2012), pluripotency (Masui et al., 2007) and reprogramming (Polo et al., 2013; Sarkar and Hochedlinger, 2013). *Sox2* null embryos die soon after implantation, masking a potential role for Sox2 in the CNS (Avilion et al., 2003; Mandalos et al., 2012). However, the function of Sox2 during neurogenesis has only recently begun to be elucidated using conditional mutagenesis in mice (Episkopou, 2005). Sox2 is expressed initially in NEPs and later in NSCs in the neurogenic niches of the embryonic and adult CNS (Wood and Episkopou, 1999; Avilion et al., 2003; Favaro et al., 2009; Remboutsika et al., 2011; Kang and Hebert, 2012; Mandalos et al., 2012; Raitano et al., 2013; Ramasamy et al., 2013). Sox2 becomes down-regulated during the final cell cycle of NEPs, immediately before differentiation and is required for the maintenance of NEPs properties functioning partly through the Shh and wnt3a pathways during embryonic and early postnatal life (Favaro et al., 2009). NSCs are completely lost from the early postnatal hippocampus upon loss of Sox2 but they gradually re-appear, an observation attributed to compensatory functions of Sox1 and Sox3 proteins, which are both expressed in an overlapping manner with Sox2. At this point, it is worth to mention that Sox2 maintains the embryonic cortical NSCs cycling, while at the same time it preserves and regulates their Pax6^+^ cortical radial identity and plasticity (Remboutsika et al., 2011; Raitano et al., 2013). Nevertheless, the exact role of Sox2 during cell cycle regulation of NSCs and NEPs is yet elusive. Recent evidence suggests that the function of SoxB1 factors, with even overlapping activities, is not strictly redundant, as they induce different sets of genes and are likely to partner with different proteins to maintain progenitor identity (Archer et al., 2011). It is reasonable then to suggest that during neurogenesis post-translational modifications, context-specific selection of partner proteins or competition for the same target binding sites modulate the activity of SoxB transcription factors. Thus, despite the discovery of SoxB transcription factors for over fifteen years their role during cell cycle, neurogenesis, neuronal commitment and specification has just started to be elucidated.

## Dedication

This review is dedicated to the memory of Prof. Larysa Pevny.
